# The Link between Landscape Characteristics and Soil Losses Rates over a Range of Spatiotemporal Scales: Hubei Province, China

**DOI:** 10.3390/ijerph182111044

**Published:** 2021-10-21

**Authors:** Qing Li, Yong Zhou, Li Wang, Qian Zuo, Siqi Yi, Jingyi Liu, Xueping Su, Tao Xu, Yan Jiang

**Affiliations:** 1 The College of Urban & Environmental Sciences, Central China Normal University, Wuhan 430079, China; ennstar@mails.ccnu.edu.cn (Q.L.); wli_gis@mails.ccnu.edu.cn (L.W.); zuoqccnu@mails.ccnu.edu.cn (Q.Z.); siqiyi@mails.ccnu.edu.cn (S.Y.); liujy21@mails.ccnu.edu.cn (J.L.); suxueping0504@mails.ccnu.edu.cn (X.S.); xutao@mails.ccnu.edu.cn (T.X.); jiangyanccnu@mails.ccnu.edu.cn (Y.J.); 2 Key Laboratory for Geographical Process Analysis & Simulation of Hubei Province, Central China Normal University, Wuhan 430079, China

**Keywords:** landscape pattern analysis, multi-scale, hotspot analysis, geodetector, Chinese soil loss equation

## Abstract

Controlling soil erosion is beneficial to the conservation of soil resources and ecological restoration. Understanding the spatial distribution characteristics of soil erosion helps find the key areas for soil control projects and optimal scale for investing in a soil and water conservation project at the lowest cost. This study aims to answer the question of how the spatial distribution of soil erosion in Hubei Province changed between 2000 and 2020. Moreover, how do the effects of natural factors and human activities on soil erosion vary over the years? What are the differences in landscape pattern characteristics and the spatial cluster of soil erosion at multiple administrative scales? We simulated the spatial distribution of soil erosion in Hubei province from 2000 to 2020 by the Chinese Soil Loss Equation model at three administrative scales. We investigated the relationship between soil erosion and driving factors by Geodector. We explored the landscape pattern and hotspots of land at different levels of soil erosion by Fragstat and hotspot analysis. The results show that: (1) The average soil erosion rate decreased from 2000 to 2020. Soil erosion is severe in the mountainous areas of western Hubei province, while it is less severe in the central plains. (2) Land-cover type, precipitation, and normalized difference vegetation index are the most influencing factors of soil erosion in 2000–2010, 2015, and 2020, respectively. (3) The aggregation index values at the town scale are higher than those at the city and county scales, while the fractal dimension index values at the town scale are lower, which indicates that soil erosion projects are most efficient when the project unit is ‘town’. (4) At the town scale, if the hotspot area (6.84% of the total area) is treated as the protection target, it can reduce 50.42% of the total soil erosion of Hubei province. Hotspots of soil erosion overlap with high erosion zones, mainly in the northwestern, northeastern, and southwestern parts of Hubei province in 2000, while the hotspots in northwestern Hubei disappear in 2020. In conclusion, land managers in Hubei should optimize the land-use structure, soil and water conservation in slope land, and eco-engineering controls at the town scale.

## 1. Introduction

Soil erosion is one of the biggest ecological problems in the world, as it leads to water pollution, reduced land productivity and water storage capacity, and deterioration of the ecological environment, which ultimately threatens human survival [1]. According to the National Soil and Water Conservation Plan (2015–2030) approved by the State Council of China, the funds will be allocated to those areas where soil erosion is severe and clustered, where a soil conservation project is urgently needed, and where the implementation of the large-scale ecological restoration project is possible [2]. To formulate land-management policies that meet local conditions and alleviate regional soil erosion, land managers and policymakers need to understand the spatial distribution characteristics of regional soil erosion and determine where soil erosion is more serious and where land with serious soil erosion is more clustered to determine the key areas for soil restoration projects. They need to understand what factors affect local soil erosion and develop targeted treatments to reduce the cost of ecological restoration [3,4,5].

Soil erosion is influenced by both natural factors and human activities, including regional topography and geomorphology, non-linearly varying rainfall erosion, soil erodibility, vegetation cover, and artificial protection measures [6,7]. Humans can influence soil erosion by changing land use and land cover [8,9]. Compared with natural influencing factors, human interference is more controllable, and understanding the main factors affecting regional soil erosion can help in developing effective ecological restoration measures [10]. Therefore, a systematic understanding of the factors influencing soil erosion over time, especially the interaction between human disturbance factors and soil erosion rates, is needed to improve soil protection and control measures [4,5,9].

Empirical models have been used to analyze the spatial distribution of soil erosion [11], with the Universal Soil Loss Equation (USLE) model or the Revised Universal Soil Loss Equation (RUSLE) model being the most widely used [12]. The modeling results can show the spatial variability of soil erosion, but the landscape heterogeneity of the spatial distribution of soil erosion should be further quantified [13]. The landscape pattern analysis method developed for landscape ecology can effectively reveal the complexity of soil erosion patterns [14]. Due to the influence of landforms on precipitation distribution and the nonlinear and stochastic nature of climate change accelerating extreme precipitation, the spatial distribution pattern of soil erosion tends to change at different scales of observation, thereby affecting the mechanism of soil erosion and its spatial and temporal variability characteristics [15,16,17].

Therefore, measuring multi-scale landscape pattern indices has important scientific significance for the rational allocation of land resources [16,18]. Some scholars have used landscape pattern indices to analyze the dynamic evolution characteristics of soil erosion areas at different scales, and an increasing number of scholars have begun to explore the multi-scale evaluation and scale effects of soil erosion [19,20]. However, most of the current studies on scale changes lack consideration of actual administrative divisions and only consider the changes of soil erosion under different scales of grid changes, while in reality, the management of human activities is based on administrative divisions and soil conservation projects are usually funded and implemented by the government on an administrative unit [21]. The results of studies based on different grid scales cannot be directly applied to land-use management planning, and few studies have been conducted to understand the spatial and temporal characteristics of soil erosion based on different administrative scales.

Identifying critical areas of soil erosion and implementing targeted management interventions can help control erosion effectively and economically [22]. Recently, hotspot analysis has been used to identify the most important areas of soil erosion and provide optimized and cost-effective management options to reduce soil erosion [23]. As soil erosion processes vary with scale, the amount of soil erosion in hotspot areas fluctuates [3]. However, the current hotspot analysis research mainly focuses on the distribution of hotspots at a single grid scale and the indication of ecosystem service protection [23]. How does the spatial distribution of soil erosion hotspot areas at different administrative scales differ? At what scales and in which areas can soil and water conservation projects be carried out at low cost and high efficiency? Few studies have answered these questions.

**Figure 1:**
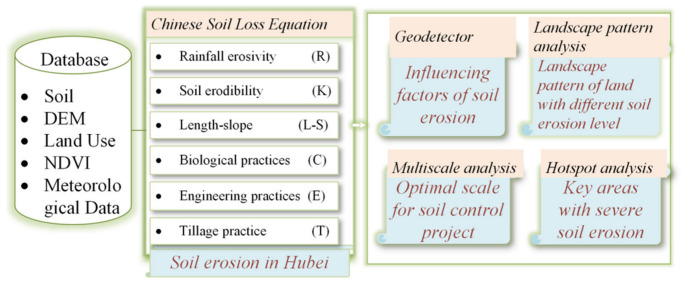
Workflow of this study.

To address the abovementioned issues, this study assessed the multi-scale spatial and temporal variation of soil erosion losses in Hubei Province in 2000, 2005, 2010, 2015, and 2020 using the Chinese version of the RUSLE model, Chinese Soil Loss Equation (Figure 1). Compared with previous studies, this study innovatively examined the scale effects of soil erosion based on different administrative scales and identified effective control types and areas by integrating landscape patterns and hotspot analysis to suggest effective erosion control in regional landscape planning. The main objectives of this study were to: (1) Reveal the spatial characteristics of soil erosion; (2) Quantify the contributions of different influencing factors; (3) Identify the optimal control units and key areas.

## 2. Study Area and Data Sources

### 2.1. Study Area

**Figure 2:**
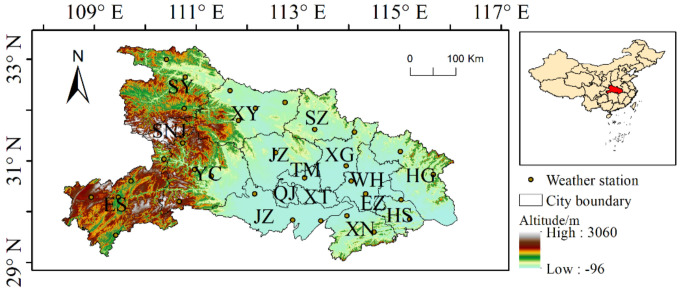
Location and altitude of Hubei province.

Hubei province is located in central China (Figure 2). Of the total area of the province, mountains account for 56%, hills for 24%, and plains and lakes for 20%. Except for the high mountain areas, most of the province has a humid subtropical monsoon climate and is the most serious area in China in terms of soil erosion, which is mainly located in northwest Hubei, southwest Hubei, northeast Hubei, and southeast Hubei. Severe soil erosion has led to the siltation of rivers, lakes, and reservoirs in Hubei, aggravating flooding, destroying land resources, reducing the productivity of arable land, deteriorating the production and living conditions of rural people, restricting economic development, increasing poverty, and making it difficult to grow land, obtain water, and increase income [24].

### 2.2. Data Sources

The data sources of this paper are described in Table 1. All the data were resampled at a 1 km × 1 km resolution.

## 3. Method

### 3.1. Chinese Soil Loss Equation (CSLE)

We used the CSLE as the following equation:

(1) A=R⋅K⋅L⋅S⋅B⋅E⋅T

where *A* is soil loss in t·ha^−1^·yr^−1^. The calculation steps of other factors in this formula are as follows. *R* is rainfall erosivity in MJ·mm·ha^−1^·yr^−1^. K is soil erodibility in t·h·MJ^−1^·mm^−1^. *L* and *S* are dimensionless topographic factors of the slope length and the slope steepness. B is the dimensionless vegetation cover factor of biological practices for trees, shrubs, and grasslands. *E* is the dimensionless factor of engineering practices. *T* is the dimensionless factor of tillage practices. The major difference between CSLE and USLE is that soil conservation practice factors of crop management (C-factor) and erosion-control (P-factor) used in the USLE are described by three erosion-control factors: biological (B-factor), engineering (E-factor), and tillage (T-factor) according to Chinese soil and water conservation classifications. Based on the modeling results, the national soil erosion classification standard table [2], and a similar study [30], the soil erosion zones with different erosion levels were classified into six categories (Table 2). See Appendix A, Appendix B, Appendix C, Appendix D, Appendix E and Appendix F for details of CSLE.

### 3.2. Geodetector

Geodetector is a statistical tool to measure the spatial stratified heterogeneity (SSH) and to explore the determinants of the spatial heterogeneity (SH). The q value of Geode-tector was used to detect how much of the SH of the soil erosion value was explained by a given factor or by two factors [31,32]. The formula of the q value is as follows:

(2) $$q=1-\frac{\sum_{h=1}^{l}N_{h}\sigma_{h}^{2}}{N\sigma_{}^{2}}=1-\frac{\sum_{h=1}^{L}\sum_{i=1}^{N_{h}}{\left(Y_{hi}-\bar{Y_{h}}\right)}^{2}}{\sum_{i=1}^{N}{\left(Y_{i}-\bar{Y_{h}}\right)}^{2}}$$

where *N* means the number of units that composed the study area; the study area is stratified into *h* = 1, 2,…, *L* stratum; stratum *h* is composed of *N_h_* units; *Y_i_* and *Y_hi_* denote the value of unit in the population and in stratum, respectively; the value range of q is [0,1]. *A* larger value of q indicates a stronger explanatory power of the independent variable *X* for attribute *Y*, and vice versa.

The input data for Geodetector for *Y* (dependent variable) are as follows: the soil erosion value, the input data for Geodetector for *X* (influencing factor) are as follows: altitude, lithology, rainfall, slope, NDVI, land-use type. We classified the continuous datasets based on the requirement of Geodetector and previous research. The altitude, rainfall, and NDVI are divided into nine strata using the natural break method. The slope was divided into grades from first to sixth: <5, 5–10, 10–15, 15–20, 20–35, and >35, respectively.

### 3.3. Landscape Pattern Analysis

We used the aggregation index (*AI*) to measure aggregation levels of soil erosion spatial patterns. AI is class-specific and independent of landscape composition, and has better performance than other landscape indices when measuring clusters of spatial patterns [33]. We used fractal dimension (*FRAC*) to represent shape aspects of patches at different soil erosion levels. In landscape ecological research, patch shapes are frequently characterized via the fractal dimension of the object. The fractal dimension index is appealing because it reflects shape complexity across a range of spatial scales [34]. We analyze landscape patterns at the class level by Fragstats 4.2 software. A brief description of the two indicators is as follows.

#### 3.3.1. Aggregation Index (*AI*)

(3) $$AI=\left[\frac{g_{\mathrm{jj}}}{\max\to g_{\mathrm{jj}}}\right](100)$$

where g_jj_ is the number of like adjacencies (joins) between pixels of patch type (class) i based on the single count method. max-g_jj_ is the maximum number of like adjacencies (joins) between pixels of patch type (class) i based on the single-count method (0 ≤ *AI* ≤ 100). Given any patch, *AI* equals 0 when the focal patch type is maximally disaggregated; *AI* increases as the focal patch type is increasingly aggregated and equals 100 when the patch type is maximally aggregated into a single, compact patch [33].

#### 3.3.2. Fractal Dimension Index (*FRAC*)

(4) $$FRAC=\frac{2\ln(0.25p_{ij})}{Ina_{ij}}$$

where *p_ij_* is perimeter (m) of patch ij, aij is area (m) of patch *ij*, 1≤ *FRAC* ≤ 2. *FRAC* approaches 1 for shapes with very simple perimeters such as squares and approaches 2 for shapes with highly convoluted, plane-filling perimeters [34].

### 3.4. Hotspot Analysis

In this paper, we used Getis–Ord $G_{i}^{*}$ statistic to find out where soil erosion with either high or low values clusters spatially. A significant hotspot is a highly soil-eroded area surrounded by other highly soil-eroded areas. The cold spot indicates a low soil erosion area surrounded by a low soil erosion area. The formula of $G_{i}^{*}$ statistic is as follows:

(5) $$G_{i}^{*}=\frac{\sum_{j}^{n}w_{ij}a_{j}}{\sum_{j}^{n}a_{j}}$$

(6) $$Z=\frac{G_{i}^{*}-E\left(G_{i}^{*}\right)}{\sqrt{Var\left(G_{i}^{*}\right)}}$$

where $G_{i}^{*}$ is the cluster index of cell *i*; *Z* is the significance of *G^∗^*; w_ij_ is the spatial weight; *a_j_* is the soil erosion value of cell j. *E*($G_{i}^{*}$) is the expectation value of $G_{i}^{*}$ and Var($G_{i}^{*}$) is the variance of $G_{i}^{*}$. The absolute value of z-score is positively correlated with the degree of cluster of cold and hotspots.

## 4. Results

**Figure 3:**
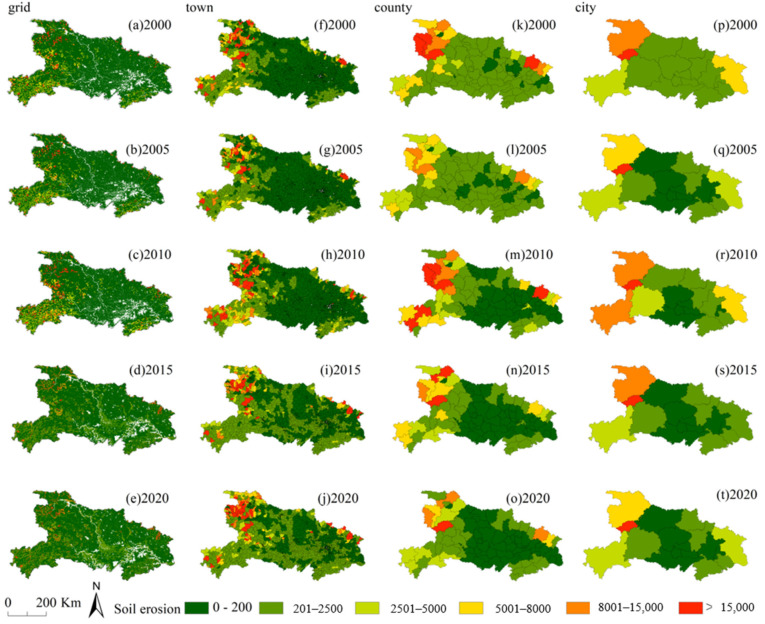
The average soil erosion rate at the (**a**–**e**) gird scale, (**f**–**j**) town scale, (**k**–**o**) county scale, and (**p**–**t**) city scale from 2000 to 2020.

The CSLE model estimated that the average soil erosion rate in Hubei was 3301.81 t·ha^−1^·yr^−1^ from 2000 to 2020 (Figure 3). Land with slight and light soil erosion levels accounted for 77% of the total area of Hubei Province. At the same time, land with severe and very high soil erosion levels covered 8.63% of Hubei and was mainly concentrated in mountainous areas. The total amount of soil erosion on severely eroded land was high, although the area of land with severe erosion was relatively small. The year with the highest average soil erosion was 2010, followed by 2020. The year with the lowest average soil erosion was 2005, followed by 2015. Spatially, from 2000 to 2020, the high, very high, and severely eroded areas in the northwestern, southwestern and northeastern regions of Hubei Province showed fluctuating trends, with the largest area in 2010.

From 2000 to 2020, soil erosion rates showed fluctuations, with the highest soil erosion in 2010 and the lowest soil erosion in 2005; the highest value was 302% of the lowest value, and the area of slight erosion increased from 42.78% in 2000 (Figure 3a) to 64.80% in 2020 (Figure 3e), and the most severely eroded area increasing from 1.52% of the total area in 2000 to 2.05%, but severe erosion declining from 8.94% of the total area to 5.17% in 2020. They all showed fluctuating changes rather than linear increases or decreases, and the area of slight erosion was the erosion class that fluctuated the most during the study pe-riod. In addition, soil erosion rates did not show dynamic changes corresponding to dif-ferences in scale. At the city, county, and grid scales, the severe, very high, and high soil erosion areas have increased from 2000 to 2010 and decreased from 2010 to 2020. The town scale (Figure 3f–j) showed a fluctuating decreasing trend from 2000 to 2005, an increasing trend from 2005 to 2010, a decreasing trend from 2010 to 2015, and then an increasing trend from 2015 to 2020.

The area of land with severe soil erosion in the plains decreased gradually, and these lands are dispersed as the scale decreased. At the city scale (Figure 3p–t), the high erosion rate areas were mainly located in the mountainous areas of western Hu-bei, of which the Shennongjia Forest Area had the highest soil erosion among the cities in Hubei Province from 2000 to 2020. In addition, the soil erosion in Shiyan city was more severe over time, mainly because the city is located in a mountainous area with steeper topographic slopes and weaker soil conservation capacity. In contrast, cities in the central and southern parts of Hubei Province have lower erosion rates than those in the northern, eastern, and western parts.

From 2000 to 2020, severe erosion zones were clustered in the southwest and northwest Hubei, mainly including Shiyan city, Enshi city, and Shennongjia Forestry District (Figure 3). Overall, soil erosion in Hubei province has shown a decreasing trend, with the emergence of various traditional and modern conservation structures leading to an increase in vegetation cover and a significant reduction in soil loss. During this period, a series of national policy measures have been taken to reduce soil erosion, one of which is the return of cultivated land to forests program. The Grain for Green project has achieved significant effects in soil erosion management. In 1999, Hubei Province started a pilot project to return farmland to forest, and in 2002, it was fully rolled out. Since 1999, Hubei has returned a total of over 1.33 × 104 km^2^ of farmland to forest, including 4193.33 km^2^ of reforestation of sloping farmland and 7240 km^2^ of reforestation of barren mountains and wastelands. The project areas are mainly concentrated in the Three Gorges Reservoir, Danjiang Reservoir and Wuling Mountains, Qinba Mountains, Dabie Mountains, and Makufu. The project area is mainly clustered in the Three Gorges Reservoir Area, Danjiang Reservoir Area and Wuling Mountain Area, Qinba Mountain Area, Dabie Mountain Area, Moufu Mountain Area, and other ecologically important areas. Until 2019, the forest coverage rate in Hubei province increased by 7 percent, and the living wood accumulation increased by more than 30 million cubic meters [35]. The ecological benefits are reflected in various aspects, such as reduced soil erosion, increased soil fertility, and reduced wind and sand erosion.

### 4.1. Factors Influencing Soil Erosion

**Figure 4:**
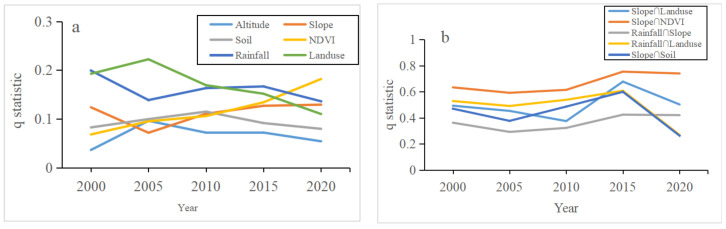
The q values of influencing factors on soil erosion from 2000 to 2020 ((**a**) q statistic; (**b**) interaction q statistic).

The factor analysis of soil erosion in Hubei Province using Geodetector showed that the height, slope, soil, NDVI, rainfall, and land use type had a significant influence on soil erosion (Figure 4a), and soil erosion was influenced by both natural and human activities. From 2000 to 2015, land use type and precipitation had a greater influence on the soil erosion rate than other factors, and land use type and precipitation had a greater impact on soil erosion rates than other factors from 2000 to 2015. In 2000, the NDVI had the second lowest influence on the soil erosion rate among the six factors; however, its influence gradually increased to become the most influential factor on the soil erosion rate in 2020. The degree of influence of land use type on soil erosion tended to fluctuate downwards and was surpassed by slope and the NDVI.

In addition, the combined effect of the two factors on soil erosion had a greater influence than that of the single element (Figure 4b). The slope and NDVI, slope and land use, rainfall, and land use all had a greater influence on the rate of soil erosion together than when they were single elements. In the future, land management policymakers should optimize the land use structure and pay attention to soil conservation projects on sloping land and other ecological greening constructions.

### 4.2. Landscape Pattern Analysis of Land at Different Soil Erosion Levels

**Figure 5:**
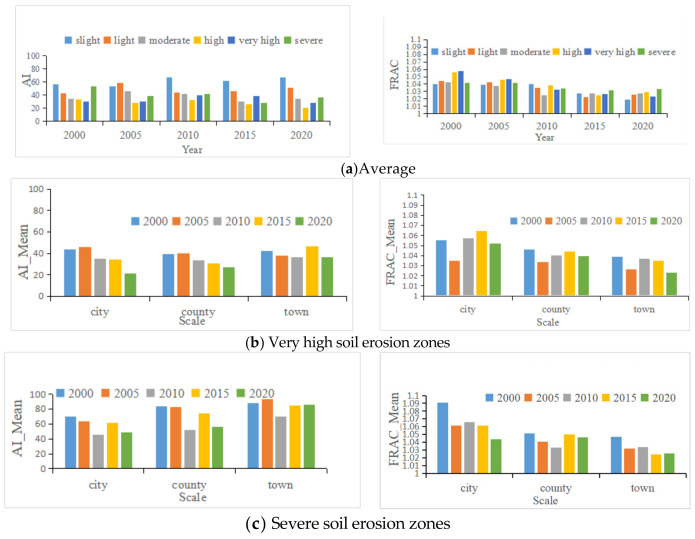
(**a**) AI and FRAC indexes of land at different soil erosion levels. (**b**) Mean value of AI and FRAC at three scales of very high erosion zones from 2000 to 2020. (**c**) Mean value of AI and FRAC at three scales of severe erosion zones from 2000 to 2020.

In terms of the interannual variation in the mean AI values of the different soil erosion zones (Figure 5a), between 2000 and 2020, the highest AI values were in the slight, light, and severe soil erosion zones. The AI values in the slight soil erosion zone and light soil erosion zone fluctuated upwards, while the AI values in the high soil erosion zone and the more se-verely eroded zones fluctuated and decreased. The AI values showed that the land with slight, light and severe erosion levels was more clustered than the land with other soil erosion levels (Figure 5a) and was concentrated in the central and eastern plains of Hubei Province; the AI values of the high and very high soil erosion zones were the lowest, indicating that the distribution of the more eroded soils became more dispersed. The lowest AI values were in the high and very high soil erosion zones, indicating that the land with higher soil erosion was scattered, mainly in the mountainous areas of western Hubei Province and the hills of eastern Hubei. This phenomenon indicates that the land with high soil erosion was relatively difficult to modify in Hubei.

The change in the average FRAC values for the different levels of soil erosion areas between 2000 and 2020 (Figure 5b) showed that the FRAC index for the light erosion zones was higher than that for the high soil erosion zones in 2000. The fluctuating FRAC values in light erosion zones decreased, while the FRAC values in severe erosion zones fluctuated and increased until 2010, when the complexity of soil patches in light erosion zones started to be lower than that in high erosion zones. The higher the FRAC index was, the more complex the shapes of the patches. Therefore, the higher the FRAC value in a patch, the more difficult it was to implement soil conservation measures. The FRAC values in the severe erosion zones were still high (Figure 5b,c). In conclusion, it is still difficult to carry out soil erosion protection projects in Hubei Province.

In terms of the average AI values at three different scales, both the AI values of the very high soil erosion zone and the AI values of the severe erosion zone were greater at the town scale than at the city scale. If the aggregation of land with serious soil erosion is low, it is difficult to concentrate soil and water conservation work, and there is no cluster bene-fit, which increases the cost of soil and water management. The FRAC values of the very high erosion areas varied greatly among the three scales, with the highest average FRAC values in cities and the lowest average FRAC values in towns, while the FRAC values of the severe erosion areas varied greatly among the three scales. Therefore, erosion control projects can be carried out on a town-by-town basis. In addition to selecting the scale for erosion project management, hotspot analysis should also be used to select key conservation sites for soil and water conservation projects.

### 4.3. Hotspot Analysis of Soil Erosion

**Figure 6:**
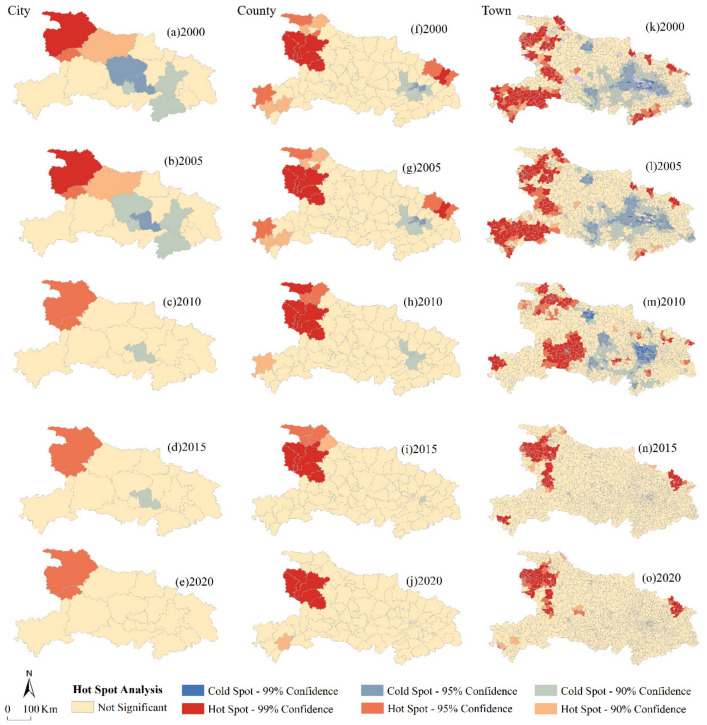
The hotspots and cold spots of soil erosion at (**a**–**e**) city scale, (**f**–**j**) county scale, and (**k**–**o**) town scale from 2000 to 2020.

The soil erosion hotspots at 99% confidence in 2000, 2010, and 2020 account for about 12.43%, 11.79%, and 5.73% of the town overall, while the 99% confidence cold spot areas account for 6.07%, 12.63%, and 0% of the town overall (Figure 6), respectively. At the city scale, hotspots were clustered in the northwestern mountainous areas, over-lapping with the severely eroded soil areas. The average rate of soil erosion in the hotspot area at the town scale with a significance of 90% and above was 8179.50 t·hm^−2^·yr^−1^, which is a very high soil erosion level. Compared with the analysis of the spatial distribu-tion characteristics of land with different soil erosion levels, hotspot analysis of soil ero-sion revealed the cluster of land with the same soil erosion level. In this study, the hotspot analysis revealed that there was an area of high soil erosion in northwestern Hubei Prov-ince. It would be more cost-effective to carry out land conservation projects in hotspot are-as. In contrast, the cold spot patches clustered in the central and southeastern plains in 2000, gradually decreased from 2000 to 2015 and disappeared by 2020. The continuity of areas with relatively low soil erosion rates compared to areas with higher erosion rates is related to the topographic characteristics and land use structure of Hubei Province. Relatively large-scale soil conservation measures need to be implemented.

Compared to the city scale (Figure 6a–d), two hotspots, the northeast and southwest, have appeared at the county scale. They both decreased gradually from 2000 to 2010. The cold spot at the town scale formed a patch in the central plains that decreased from 2000 to 2015 and disappeared in 2020. It is worth noting that this cold spot area did not overlap with the cold spot area at the city scale.

The hotspot area at the town scale included not only the northwestern and south-western mountainous areas but was also scattered in the northeastern and southeastern mountainous areas, decreasing from 2000 to 2020 (Figure 6k–o). The cold spots were located in the central, southern, and eastern plains. From 2005 to 2020, the hotspot areas gradually decreased, and the hotspot distribution map showed a trend towards fragmentation. The distribution of high soil erosion areas became dispersed. The average soil erosion rates in the hotspot areas decreased at both the county and town scales, with decreases of 53.68% and 43.92%, respectively. During the study period, guided by the Chinese central government’s soil and water conservation policies and program, the Hubei provincial government actively participated in the Grain for Green program [36,37], a which may slow soil erosion in Hubei. As the scale changed from the county scale to the town scale, land with high soil erosion rates in southwestern Hubei Province became dis-persed hotspots in 2010.

The hotspot area at the city scale was only 84.29% of the hotspot area at the town scale, but the average soil erosion rate of soil erosion hotspots in Hubei Province at the town scale was 12.17% higher than that at the county scale. The hotspots at the town scale were more clustered and severe, making soil conservation measures less costly and more effective. For town-scale hotspot areas (6.84% of the total area of Hubei Province in 2020), 50.42% of the total soil erosion was reduced. Town-scale hotspots were mostly located in the mountainous and hilly areas of Hubei Province. To control soil erosion in town-scale hotspots, land managers should combine erosion control projects with ecological restora-tion projects on sloped land to optimize the regional ecological layout [38].

## 5. Discussion

### 5.1. Comparison with Existing Studies

**Figure 7:**
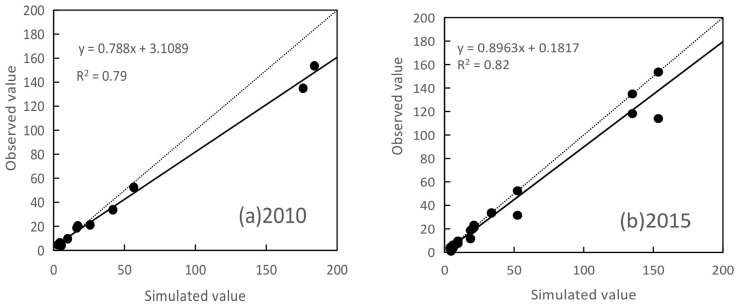
Correlation analysis between simulated value (10^5^ t) and observed value (10^5^ t) in (**a**) 2010 and (**b**) 2015.

We found that the areas of severe soil erosion in Hubei Province were mainly in the mountainous areas of western Hubei Province, including Enshi, Shiyan, and Yichang, which is consistent with the results of Zeng’s research [39]. This indicates that the western mountainous areas of Hubei Province have been the areas with high soil erosion from 1980 to the present. By comparing the remote sensing survey data of soil erosion in Hubei Province, 52.41%, 56.17%, and 77.76% of the total eroded area in 2006, 2011, and 2019 in Hubei Province [24], it can be seen that soil erosion is on a decreasing trend, and all these trends are consistent with the results of this paper. Our study shows that the soil erosion area in Hubei province decreases, with light erosion accounting for 42.79%, 53.61%, and 77.02% of the total erosion area in 2005, 2010, and 2020(Figure 7). However, there are differences in the evaluation results because of different evaluation methods and different evaluation years.

We found that soil erosion in Hubei Province is more correlated with land use, slope, and vegetation cover. During the study period, key projects for soil erosion control in Hubei Province include the National Key Construction Project of Soil and Water Conservation, the Comprehensive Management Project of Soil and Water Erosion on Sloping Arable Land, the Soil and Water Conservation Project in Danjiangkou Reservoir Area and Upstream, the Soil and Water Conservation Project for Consolidating the Results of Returning Cultivated Land to Forests, and the Soil and Water Conservation Project for Comprehensive Management of Rock Desertification in Karst Areas [24]. These ecological restoration projects reduce soil erosion in Hubei Province by increasing vegetation cover and reducing erosion loss through grass retention and tree planting, and reducing the topographic slope of the area to reduce the flow rate of water on the slope and weaken the erosion effect of flowing water on the ground. This analysis is similar to the findings of Xiao Wang et al.’s research [40].

Due to the lack of erosion data in the region, the soil erosion results estimated by the CSLE model in this study were analyzed using the observations of river sand transport monitoring stations at the outlet of each watershed of typical rivers (11 river sand transport monitoring stations in 2010 and 2015) from the Hubei Soil and Water Conservation Bulletin for correlation analysis. The results showed that the overall correlation coefficient was 0.79 in 2010 and 0.82 in 2015, indicating that the soil erosion estimation results for these two years were satisfactory. This could partially reflect the accuracy of the soil erosion results. Besides, most of the existing studies on soil erosion in China are on the Loess Plateau in northern China [30], the Beijing–Tianjin–Hebei region [41], and the Yunnan–Guizhou Plateau in southern China [20,42,43], but few studies on the spatial distribution characteristics, landscape patterns, and influence mechanisms of soil erosion have been conducted in central China. Few studies are available for comparison of soil erosion in Hubei province.

### 5.2. Methodological Limitations

Because of the inconsistent resolution of precipitation data, DEM data, soil data, LULC data, and NDVI data, the accuracy of the calculation results are relatively rough. Besides, the R-factor could be calculated in various ways due to the different data resources available in different locations, based on annual precipitation data [44], monthly precipitation data, and daily precipitation data, and it is difficult to compare the results of similar research with similar topics since different calculations are used for soil erosion analysis [45,46,47]. As high-resolution data increases, future research should use a higher resolution to reduce calculation errors.

## 6. Conclusions

In this study, the multi-scale spatial-temporal variations in soil erosion loss in 2000, 2005, 2010, 2015, and 2020 were evaluated using the CSLE model in Hubei Province. In contrast to previous studies, this study innovatively examines the effect of soil erosion characteristics based on different administrative scales and identifies effective control types and regions by integrating landscape patterns and hotspot analyses to propose effective soil erosion control suggestions in regional landscape planning. The results show that soil erosion fluctuated in Hubei over the study years. Scaling effects existed in the spatial characteristics of soil erosion. The landscape pattern and hotspots of soil erosion in each year changed with scale. The slope, NDVI and land use had a greater influence on the rate of soil erosion than other factors. The impacts of human activities increased over time and as the scale decreased. The town scale was the best control scale based on the scale effect analysiss.

The sustainability of human societies depends on the wise use of natural resources. Soils contribute to basic human needs. To make regional policies and plans for soil conservation, it is necessary to identify where the soil erosion problems are, which means knowing where soil erosion rate is exceeding the soil loss tolerance. In the future, researchers could explore the trade-offs between soil erosion and other ecological services at multiple scales, which would be valuable references for effective and sustainable management and policy decisions for minimizing trade-offs and maximizing synergies of ecological services. Land managers in the Hubei government need to optimize the land-use structure, pay attention to sloping land improvement [48], increase vegetation coverage [49,50], and increase ecological restoration projects in the west of Hubei. Besides, land policymakers should consider the scale effect of soil and water conservation projects when making land-use plans.

## Figures and Tables

**Table 1:** Data description.

Data Name	Data Source	Time	Units/Resolution
Depth to bedrock map of China	Scientific data [25]	2018	100 m × 100 m
Soil types	Harmonized World Soil Database (HWSD) [26]	2012	1:1,000,000
Land-use/land cover data	Resource and Environment Science and Data Center [27]	2000; 2005; 2010; 2015; 2020	30 m × 30 m
Normalized difference vegetation index (NDVI)			1000 m × 1000 m
Meteorological data	Meteorological Data Center of China Meteorological Administration [28]	2000–2020	Daily
Digital elevation model (DEM)	Shuttle Radar Topography Mission (SRTM) [29]	2008	30 m × 30 m

**Table 2:** The standard for soil erosion level.

Soil Erosion Level	Slight	Light	Moderate	High	Very High	Severe
Soil erosion rate (t·ha^−1^·yr^−1^)	<200	200–2500	2500–5000	5000–8000	8000–15,000	>15,000

## Data Availability

Not applicable.

## Appendix A. Rainfall Erosivity Factor (R)

The equations of average annual rainfall erosivity and 24 ratios of half month erosivity to annual erosivity calculation using daily erosive rainfall data were represented as follows by referring Liu’s research [51].

(A1) $$R_{\mathrm{year}}=\sum_{j=1}^{24}R_{j}$$

(A2) $$Rj=\frac{1}{N}\sum_{i=1}^{N}\sum_{k=0}^{m}\left(\alpha \cdot P_{i,j,k}^{{}^{1.7265}}\right)$$

(A3) $$RR_{j}=R_{j}/R_{year}$$

where *R_year_* is average annual rainfall erosivity in MJ·mm·ha^−1^·h^−1^·yr^−1^, j represents the half month sequence of 1, 2, …, 24 ineach year, *R_j_* is half month rainfall erosivity in MJ·mm·ha^−1^·h^−1^, i is time series of daily rainfall from 1981 to 2010, k is days of daily erosive rainfall (rainfall equal or greater than 10 mm) within each half month period, *P_i,j,k_* is 0 when no erosive rainfall occurs in the half month, and a represents calibrated parameter values of 0.3957 for warm months from May to September and 0.3101 for cool months from October to April. RR_j_ is the ratio of the average erosivity of half month j to the average annual erosivity. The annual rainfall erosion value was interpolated using the Kriging interpolation method in Arcgis 10.7 software to obtain the R map in Hubei province.

## Appendix B. Soil Erodibility Factor (K)

The equations of K factor (t·h·MJ^−1^·mm^−1^) in this study was referred Williams et al.,’s [52]

(A4) $$\begin{array}{c} K=0.1317\times \{0.2\\ +0.3\times \exp\left[-0.256\times \mathrm{SAND}\left(1-\frac{\mathrm{SILT}}{100}\right)\right]\}\times {\left(\frac{\mathrm{SILT}}{\mathrm{SILT}+\mathrm{CLAY}}\right)}^{0.3}\\ \times \left[1-0.25\times \frac{\mathrm{TOC}}{\mathrm{TOC}+\exp\left(3.72-2.95\times \mathrm{TOC}\right)}\right]\\ \times \left[1-0.7\times \frac{{\mathrm{SN}}_{1}}{{\mathrm{SN}}_{1}+\exp\left(22.9\times {\mathrm{SN}}_{1}-5.51\right)}\right] \end{array}$$

where, SAND, SILT, CLAY, TOC is the content for sand, silt, clay, and organic content of soil(%), ${\mathrm{SN}}_{1}=\mathrm{SAND}/100$.

## Appendix C. Length-Slope Factor (LS)

L and S are dimensionless topographic factors [53].

(A5) LS=L×S

where L is slope length, $L={\left(\frac{\gamma }{22.1}\right)}^{\alpha }$, $\alpha =\beta /\left(1+\beta \right)$,$\beta =\frac{\sin\theta /0.089}{3\times {\sin\theta }^{0.8}+0.56}$, θ is the slope value derived by DEM using Slope function ArcGIS 10.7, α is the slope exponent and β is the ratio of fine gully erosion to surface erosion [54], S is slope steepness, the calculation formula is as following.

(A6) $$S=\left\{\begin{array}{cc} 10.8\sin\theta +0.03 & \theta <5^{{}^{\circ}}\\ 16.8\sin\theta -0.05 & 5^{{}^{\circ}}\leq \theta <10^{{}^{\circ}}\\ 21.9\sin\theta -0.96 & 10^{{}^{\circ}}\leq \theta \end{array}\right.$$

## Appendix D. Vegetation and Biological Practice Factor (B)

The calculations of vegetation and biological practice factor B was referred Xie’s research. The value of B factor is based on Fraction Vegetation Coverage (FVC) (Table A1). FVC was derived based on Wang’s research [55] and Li’s research [56], and different B values were assigned to the land-use types under different vegetation cover degrees. Since bare land in the study area is very few (only 0.409%), we did not take them into the calculation.

(A7) $$\mathrm{FVC}=\left(\mathrm{NDVI}-{\mathrm{NDVI}}_{\min}\right)/\left({\mathrm{NDVI}}_{\max}-{\mathrm{NDVI}}_{\min}\right)$$

where FVC is the vegetation cover, the NDVI value with a cumulative frequency of 0.5% is the NDVI_max_ value, and the NDVI_min_ value with a cumulative frequency of 0.5% is the NDVI_min_ value.

**Table A1:** Value for B factor under different FVC value of different land cover in Hubei province.

FVC	Forestland	Grassland	Arable Land	Water Bodies	Built-Up Area
0–20%	0.1	0.45	0.23	1.0	0.9
20–40%	0.08	0.24			
40–60%	0.06	0.15			
60–80%	0.02	0.09			
80–100%	0.004	0.043			

## Appendix E. Engineering Practices (P)

P factor is determined as the ratio between the soil losses expected for a certain soil conservation practice. Without engineering practices recorded, the values were taken by refereeing Xu’s studies [57] (Table A2).

**Table A2:** Value for P factor under different land cover.

	Forestland	Grassland	Arable Land	Water Bodies	Built-Up Area
P factor	1	1	0.4	0	0

## Appendix F. Tillage Practice (T)

The criteria of tillage practice indicator (T value) was referred Liu’s research [51] (Table A3).

**Table A3:** T factor value under different slope level.

**Slope**	0–5°	5–10°	10–15°	15–20°	20–25°	>25°
**T Value**	0.100	0.221	0.305	0.575	0.735	0.800

The values at different administration levels were extracted using the zonal statistics function in spatial analysis tool of ArcGIS 10.7 software.
